# Dose-related effects of vitamin D on immune responses in patients with clinically isolated syndrome and healthy control participants: study protocol for an exploratory randomized double- blind placebo-controlled trial

**DOI:** 10.1186/1745-6215-14-272

**Published:** 2013-08-27

**Authors:** Karen O’Connell, Siobhan Kelly, Katie Kinsella, Sinead Jordan, Orla Kenny, David Murphy, Eric Heffernan, Risteard O’Laoide, Donal O’Shea, Carmel McKenna, Lorraine Cassidy, Jean Fletcher, Cathal Walsh, Jennifer Brady, Christopher McGuigan, Niall Tubridy, Michael Hutchinson

**Affiliations:** 1 Department of Neurology, St Vincent’s University Hospital, Elm Park, Dublin 4, Ireland; 2 Consultant Ophthalmologist and Occuloplastic Surgeon, Royal Victoria Eye and Ear Hospital, Adelaide Road, Dublin 2, Ireland; 3 Schools of Medicine and Biochemistry and Immunology, Trinity Biomedical Sciences Institute, Pearse Street, Trinity College Dublin, Dublin 2, Ireland; 4 Department of Statistics, Trinity College Dublin, Dublin 2, Ireland; 5 Department of Biochemistry and Diagnostic Endocrinology, Mater Misericordiae University Hospital, Eccles St, Dublin 7, Ireland; 6 Department of Radiology, St Vincent’s University Hospital, Elm Park, Dublin 4, Ireland; 7 Department of Endocrinology, St Vincent’s University Hospital, Elm Park, Dublin 4, Ireland; 8 Pharmacy Department, St Vincent’s University Hospital, Elm Park, Dublin 4, Ireland

**Keywords:** Vitamin D, Clinically isolated syndrome, Multiple sclerosis, Immunomodulation, Cholecalciferol, Clinical trial

## Abstract

**Background:**

There is increasing evidence linking vitamin D deficiency to both susceptibility to, and severity of, multiple sclerosis (MS). Patients with the clinically isolated syndrome represent the initial presentation of a demyelinating disorder, and those with asymptomatic lesions on magnetic resonance imaging (MRI) are at risk of progression to clinically definite MS. The aims of this study are to examine the immunologic effects of vitamin D in both healthy individuals and in patients with clinically isolated syndrome, and in the latter group the effects on disease progression assessed by MRI and clinical measures.

**Methods/Design:**

This is a single-center double-blind randomized placebo-controlled clinical trial. The primary endpoint is the immunologic effects of two doses of vitamin D compared with placebo over 24 weeks in both healthy control participants and patients presenting with the clinically isolated syndrome. Healthy control participants (n = 39) and patients with clinically isolated syndrome (n = 45) will be randomized to one of three arms, namely 1) vitamin D 5,000 IU daily, 2) vitamin D 10,000 IU daily, or 3) placebo, and followed up for 24 weeks. In both patients and healthy control participants, the primary outcome will be immunologic measures of the frequency of CD4 T-cell subsets and cytokine responses in peripheral blood mononuclear cells, assessed at baseline, and after 16 and 24 weeks of treatment. Secondary endpoints, in the patients with clinically isolated syndrome, will be relapse activity, and the number of new T2 lesions and gadolinium-enhancing lesions assessed by MRI in the two vitamin D-treated groups compared with the placebo-treated group over the 24 weeks of the study.

**Trial registration:**

EU Clinical Trials Register: EudraCT: 2012-000635-68. ClinicalTrials.gov identifier: NCT01728922

## Background

Multiple sclerosis (MS), a chronic inflammatory disease of the central nervous system, is the most common disabling neurologic disorder of young adults. The causes of MS remain unknown, but it is widely believed that there is interplay between genetic and environmental factors [1]. Evidence for the role of the environment in the development of MS is supported by a strong correlation between MS incidence/prevalence and increased latitude and lower levels of sun exposure, suggesting an association with vitamin D production [2-4]. Individuals who emigrate before the age of 15 years from an area of high MS prevalence to one of low prevalence, acquire the lower risk of MS in their new country of residence. Migration studies provide perhaps the strongest support for the role of environmental factor(s) in this disease [5,6].

People living at high latitudes are generally deficient in vitamin D, as measured by serum 25-hydroxyvitamin D (25(OH)D) levels [7,8]. In one study, mean winter 25(OH)D levels in healthy control subjects in Ireland were 36.4 nmol/l, and more patients with MS had vitamin D deficiency (serum 25(OH)D levels less than 25 nmol/l) compared with control subjects [9]. Patients have lower serum vitamin D levels during MS relapses, and also have blunted parathyroid hormone responses [10,11]. In 267 Dutch patients with relapsing remitting multiple sclerosis (RRMS), higher vitamin D levels were associated with an increased chance of remaining relapse-free [11]. In 134 North American patients with RRMS or clinically isolated syndrome (CIS) of pediatric onset, the strongest predictor of further relapse was the baseline serum vitamin D level [12]. In a prospective Australian study of 145 patients with RRMS, each increase of 10 nmol/l in baseline serum vitamin D level was associated with a 12% reduction in the risk of a further relapse [13]. Vitamin D deficiency may increase susceptibility to MS even *in utero*; higher maternal vitamin D intake during pregnancy was associated with a 38% lower risk of MS in offspring [14], and maternal vitamin D levels greater than 75 nmol/l were associated with a 61% reduced risk of MS in children [15]. Month of birth studies showing an increased MS incidence in those with spring versus autumn births also provide evidence of the role of maternal vitamin D [16].

The potential therapeutic effect of vitamin D supplementation was first suggested by Goldberg in 1986 [17]. Dosing studies in healthy volunteers have shown that supplementation with vitamin D 10,000 IU daily results in an increase in serum 25(OH)D levels of 150 nmol/l over a 12 week period [18]. A Canadian phase I/II open-label, randomized, placebo-controlled trial (RCT) of 49 patients with RRMS who were randomized to placebo or escalating doses of vitamin D over a 52 week period showed a trend toward relapse reduction in the actively treated group [19]. This study was primarily aimed at establishing the safety and tolerability of high-dose vitamin D supplementation; the mean vitamin D dose was 14,000 IU per day, and there were no adverse events or hypercalcamia despite maximum mean vitamin D levels of 413 nmol/l being achieved. The results from this phase II trial [19], and from dosing [18] and observational studies [12,13], suggest that pharmacologic doses of vitamin D of up to 10,000 IU/day are safe, and may reduce the risk of relapse by a factor superior to the 30% reduction reported from the pivotal trials of interferons in RRMS [20,21].

Vitamin D modulates both the innate and adaptive immune systems [22]. The active metabolite, 1,25 (OH) vitamin D, primarily mediates its effects through the intracellular vitamin D receptor (VDR) [23], which is present in monocytes, dendritic cells, B-cells, and CD4+ T-cells [11,22]. Activation of VDR alters transcription, proliferation, and differentiation of immune cells [23], and modulates immune response both indirectly, by reducing the activation of pro-inflammatory T-cells by antigen-presenting cells [24], and directly, by inhibiting T-cell and B-cell proliferation [22,25]. This results in a T-helper (Th) 2-cell driven anti-inflammatory state [11,22,26].

There is thus accumulating evidence that vitamin D deficiency increases susceptibility to MS, and that vitamin D supplementation reduces disease activity by immunomodulatory mechanisms. It seems probable that serum vitamin D levels of greater than 100 nmol/l are required to produce the immunologic effects of vitamin D and thus, in patients with MS with vitamin D deficiency, doses of 5,000 to 10,000 IU/day are needed to achieve this. Most patients with RRMS, once diagnosed, commence treatment with first-line disease-modifying therapies (DMTs). Several RCTs are underway to examine the effects of vitamin D supplementation in patients with RRMS already receiving first-line DMTs, using clinical and MRI outcomes [27,28]. In the UK and Ireland, patients with CIS are not usually commenced on DMTs until there is clinical or MRI evidence of dissemination in time. These CIS patients, at risk of developing RRMS, may be recruited into trials of potentially therapeutic agents in order to examine the efficacy of specific therapies in preventing the development of clinically definite MS [29]. We have therefore designed this RCT to examine, over a 24 week treatment period the effects on immunologic measures as a primary outcome, both in patients with CIS and in healthy control participants, randomized to either of two doses of vitamin D (5,000 or 10,000 IU) or to placebo. In addition, in the patients with CIS, clinical and MRI efficacy measures will be assessed as secondary outcomes.

## Methods/Design

### Trial design

This is a single-center investigator-led, phase II, exploratory, double-blind, dose-ranging, randomized, placebo-controlled trial to assess the immune responses to two doses of vitamin D in patients with CIS and in healthy control participants. Recruitment commenced in November 2012. In total, 45 patients with CIS and 39 healthy control participants will be enrolled and randomized to one of three arms, placebo oil or vitamin D oil (Vigantol®; Merck Serono, Darmstadt, Germany) at a dose of 5,000 IU or 10,000 IU, all of which will be taken orally for a period of 24 weeks.

### Ethics

Ethical approval has been granted by St Vincent’s Hospital Ethics and Medical Research Committee, and the study has been approved by the Irish Medicines Board. The study will comply with the ethical principles as set down in the Declaration of Helsinki, and will be conducted in accordance with good clinical practice as defined by the International Conference on Harmonisation (ICH). The trial is registered with ClinicalTrials.gov NCT01728922 and EU Clinical Trials Register EudraCT number 2012-000635-68.

### Study participants

Study participants will be composed of two groups: 1) 45 patients with CIS, and 2) 39 healthy control participants.

#### Inclusion criteria

1) Patients with CIS aged between 18 and 55 years will be eligible for entry into the study if symptom onset occurred within 3 months of screening, they have two or more asymptomatic T2 lesions on MRI brain consistent with demyelination, not treated with steroids within 30 days of screening and are not on any other DMT.

2) Healthy control participants will be recruited from hospital staff, and will be aged between 20 and 40 years with a female:male ratio of 2:1, in line with the gender ratio seen with CIS.

#### Exclusion criteria

Exclusion criteria both for patients with CIS and for healthy control participants include: no history or evidence of hypercalcemia, renal impairment, vitamin D intolerance, parathyroid dysfunction, sarcoidosis, prior or current treatment with thiazide diuretics, or vitamin D supplementation of greater than 1,000 IU/day. Exclusion criteria for patients with CIS include: 1) if their symptoms might be explained by another diagnosis other than MS; 2) occurrence of an exacerbation less than 6 weeks prior to study entry; 3) previous treatment with β-interferons or glatiramer acetate in the 3 months before study entry, previous treatment with steroids in the 4 weeks before study entry, or any previous treatment with mitoxantrone or other immunosuppressant; and d) concurrent enrolment in any other clinical trial investigating DMT in CIS.

All female patients and healthy controls will be advised not to become pregnant for the duration of the study, and if sexually active to use effective contraception for the duration of the study and for 3 months after.

### Randomization

Patients with CIS and healthy control participants who fulfill the inclusion criteria will be enrolled in the study after providing informed consent at the screening visit. At the baseline visit, study participants who remain eligible will be assigned a number in ascending order: 1 to 45 for the CIS group and 1 to 39 for controls. The CIS group will be denoted by an X before this number, and the controls a Y. These numbers were randomized using the software program GraphPad [30] by the hospital pharmacist. The healthy control participants and patients with CIS will be randomized separately to maintain equal numbers in the three treatment arms of both these study groups.

### Interventions

#### Active therapy

The active therapy is vitamin D. The active principle will be cholecalciferol in the form of an oil (Vigantol Oil®; Merck Serono, Darmstadt, Germany), which is biologically inactive and is a precursor for the metabolically active metabolite calcitriol (1,25(OH)2D3).

#### Placebo therapy

A placebo oil, not containing vitamin D but identical to the active principle in all other respects, will be used.

#### Dispensing

Both the active and placebo oils will be dispensed in bottles with droppers, which contain 10 ml of fluid, with 20,000 IU of cholecalciferol per ml.

### Study endpoints

The primary endpoint is the effect of oral vitamin D at 5,000 and 10,000 IU/day compared with placebo on the frequency of CD4 T-cell subsets and cytokine responses in peripheral blood mononuclear cells in patients with CIS and in healthy control participants at weeks 16 and 24 compared with baseline.

Secondary endpoints include: the following. 1) In the patients with CIS having MRI brain scans at baseline and week 24, the number of new and enlarging T2 lesions and gadolinium-enhancing lesions at week 24 compared with baseline in patients receiving oral vitamin D at 5000 IU and 10,000 IU/ day compared with placebo, 2) In the patients with CIS, the effects of oral vitamin D at 5,000 and 10,000 IU/day compared with placebo on relapse occurrence, calculated as the annualized relapse rate (ARR), the percentage of patients who are relapse-free, and time to first relapse. 3) The effects of oral vitamin D at 5,000 and 10,000 IU/day compared with placebo on the percentage of treated patients with CIS who are free from any evidence of disease (clinical and MRI) activity.

### Study visits and outcome parameters

**Figure 1:**
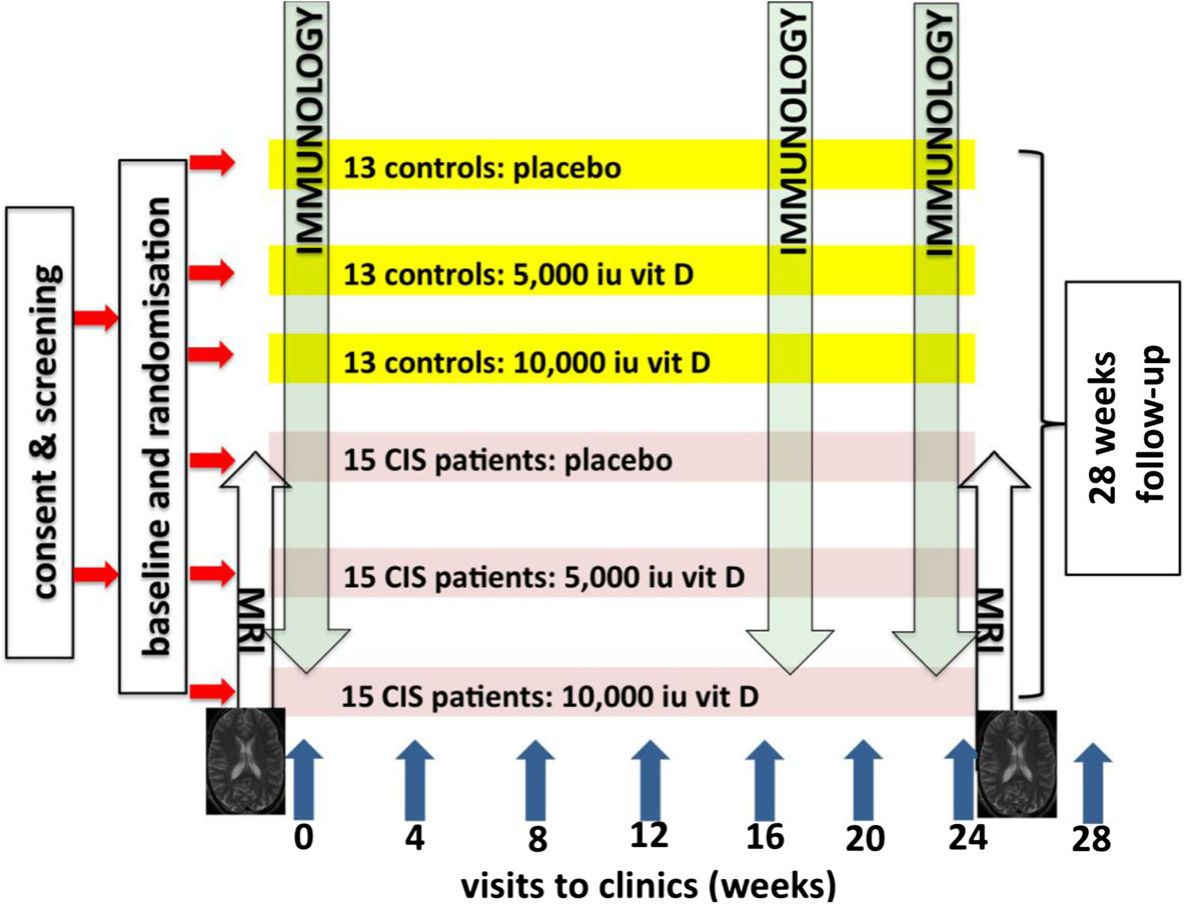
**Schematic diagram of study structure.** There are two study groups of subjects: 39 healthy control participants and 45 patients with clinically isolated syndrome (CIS). Each of the two groups will be randomly allocated to one of three blinded treatments: placebo, vitamin D 5,000 IU, or vitamin D 10,000 IU daily. Study duration is 24 weeks with immunologic testing (primary endpoint) of all study participants at baseline, week 16, and week 24. There will be 4-weekly clinic visits to assess safety measures and clinical status. 4 weeks after completion of the study there will be a follow-up safety visit. Magnetic resonance imaging will be carried out at baseline and week 24 in the CIS population only.

Participants will be screened following discussion of the trial and provision of signed, fully informed consent. If the participants satisfy the inclusion criteria at screening, they will then be scheduled for a baseline visit. The study scheme is outlined in Figure 1. There will be follow-up visits scheduled every 4 weeks visits until the end of study visit at week 24, and a safety visit at week 28 will also be arranged.

At all visits, study participants will have any changes in medical history or medications recorded, and will undergo a general physical and neurologic examination. Patients with CIS will be assessed for any evidence of relapse, defined as the appearance of a new neurologic abnormality or reappearance of a previously seen neurologic abnormality, separated by at least 30 days from the onset of the preceding event. The abnormality must be present for at least 24 hours, must be based on objective clinical evidence, and must occur in the absence of fever or known infection.

Blood biochemistry, including liver function tests, electrolytes, urea, creatinine, and serum calcium will be measured at all study visits.

Serum vitamin D levels and parathormone levels will be assessed at baseline, and at weeks 4, 8, 12, 16 and 24. Serum 25(OH)D levels will be measured by liquid chromatography-mass spectrometry. Intact parathormone levels will be measured by a two-step sandwich immunoassay using chemiluminescence technology.

Immunologic analysis will be carried out at baseline, and at weeks 16 and 24. Peripheral blood mononuclear cells (PBMCs) will be isolated within 12 hours of drawing blood, and cryopreserved until samples from all three time points have been collected. Cytokine production will be measured by ELISA, and will include 1) cytokine production by T cells and by other cells such as monocytes, and 2) flow cytometry, gating specifically on CD4 and CD8 T cells. 1) For analysis of PBMC cytokine production, PBMCs from each timepoint will be thawed out and stimulated with antigen (purified protein derivative (PPD) and tetanus toxoid) or polyclonal stimuli (anti-CD3 and phytohaemaglutinin). After 3 to 5 days (for polyclonal or antigen-specific stimulation respectively), the cell culture supernatants will be assayed by ELISA for cytokines including interleukin (IL)-10 and IL-17, and interferon (IFN)-γ. 1) For flow cytometry analysis of T-cell subsets, stimulated PBMCs from experiment 1, will be re-stimulated on day 5 with PMA and ionomycin in the presence of brefeldin A for 5 hours, followed by cell surface staining for T-cell markers (CD3, CD8) and intracellular staining for cytokines including IL-10, IL-17, and IFN-γ. The cells will be acquired on a FACSCanto II flow cytometer (Becton Dickinson), and analyzed using FlowJo software. Cytokine production by CD3+ CD8− (CD4 T-cells) and CD3+ CD8+ (CD8 T-cells) will be analyzed.

### *MRI scanning* at baseline visit and week 24

Brain MRI will be performed with contiguous axial 3 mm thick slices. Sequences will include axial and coronal T1-weighted spin echo (before and after gadolinium contrast enhancement), axial and sagittal PD/T2-weighted, and axial fast fluid attenuated inversion recovery, before contrast. MRI assessment will include 1) number of gadolinium enhancing lesions, 2) number of new and enlarging T2 lesions, and 3) the total number of new lesions (the sum of 1) and 2)).

An enhancing new T2 lesion will be considered a single lesion. Two radiologists will together examine the MRI scans at baseline and week 24, without having knowledge of the treatment group to which the patient/participant belongs. The two radiologists will determine by consensus for each study subject the numbers of new and enhancing T2 lesions at week 24 weeks compared with baseline, and thus the cumulative total number of new lesions over the 24 week period. Mean and median T2 and gadolinium enhancing lesions will be assessed for each treatment allocation group at baseline and 24 weeks.

### Rescue therapy

In the event of a relapse, the patient will undergo appropriate investigations and be commenced on steroid therapy if appropriate. As these patients will then meet the McDonald 2010 criteria [31] for diagnosis of MS, they will be offered treatment in line with the current recommendations for first-line treatment of RRMS. They will be advised to continue study visits for the duration of the trial.

Participants with CIS whose MRI scans show new lesions at the end of the study period (24 weeks) meeting the McDonald 2010 criteria [31] for MS will also be offered first-line therapy for RRMS, as is standard practice in our institution.

### Blinding

Patients, healthy control participants, and study staff, with the exception of the hospital pharmacist and internal monitor will remain blinded until the study database is locked at the end of the trial. The study drug (Vigantol®) and the placebo oil are identical in appearance, as is the labeling and packaging. Each bottle will have a unique four-digit product code, which has been generated by the hospital pharmacist, and ranges from 1000 to 2499. These numbers have been shuffled into random order, with the first group of 750 numbers assigned to the active product (Vigantol®**)** and the second group of 750 numbers to the placebo oil. Participants will be dispensed two identical bottles at 4-weekly intervals, which will be either two bottles of the study drug (Vigantol®), two bottles of placebo oil, or a bottle of each, equating to the two doses of vitamin D and placebo being investigated in this study. Participants will be advised to take a set dose from each bottle per day. Mechanisms are in place so that the code can be broken in the case of an emergency where it is essential for the clinical management of the subject.

### Sample size

The power calculations for this study were based on a pilot study, which examined immunologic outcomes in four healthy control participants treated with vitamin D 5,000 to 10,000 IU/day at our institution [32]. All calculations for numbers needed in the study were based on α = 5% and β = 80%. Based on flow cytometry analysis of T-cell subsets, the change in IL-17-producing CD4 T-cells (Th17 cells) had an effect size (mean ± SD) of 1.9 ± 1.8 indicating a need for five study subjects per group for 80% power. For IFN-γ-producing CD4 T-cells (Th1 cells), the effect size was 4.3 ± 6.3) indicating a need for 19 subjects per group for 80% power. The changes noted in cytokines in response to anti-CD3 stimulation of PBMC were 1) for IL-17, Δ = 44.5 ± 44.3, thus 10 participants needed in each group; 2) for IFN-γ, Δ = 32.8 ± 1.2, thus 6 needed in each group; and 3) for IL-10, Δ = −455 ± 27, thus number needed is < 5. The changes noted in cytokines in response to PPD stimulation were 1) for IFN-γ, Δ = 1860 ± 1835, thus 10 needed, in each group and 2) for IL-10, Δ = −576 ± 86, thus number needed is <5. On the basis of these results, at least 10 subjects should be included in each group (the only immunologic endpoint not powered would be the flow cytometry analysis of the IFN-γ-producing cells). Recruiting 39 healthy controls and 45 patients with CIS will allow for a drop-out rate of 20% and 30% respectively.

### Statistical methods

All analyses assume a two-sided test of hypothesis with an overall α level of 0.05. Immunologic endpoints and *post hoc* analyses, which will include the 16 and 24 week endpoints, are considered exploratory, and will not be adjusted for multiple comparisons. Descriptive statistics will be presented as mean ± SD. Non-parametric tests will be used to compare characteristics between treatment groups. For outcomes, the sign/sign rank test will be used to evaluate continuous outcomes. McNemar testing will be used to categorize categorical and binary outcomes. For non-parametric testing on unpaired continuous data, the Wilcoxon rank sum test will be used. All outcomes will be adjusted for baseline serum 25(OH)D levels, and for mean serum levels achieved at weeks 16 and 24.

Missing data will be imputed as the mean of the preceding and following measurements. Only patients completing at least 4 months of full participation in the study or experiencing a relapse at any time in the study will be included in the final analysis. Non-adherent participants, defined as those with an increase in serum level of 25(OH)D of less than 50 nmol/l from baseline, will be excluded from the final analysis.

### Funding

The costs of conducting the clinical trial are funded by the Department of Neurology, St Vincent’s University Hospital.

### Study sponsor

The trial sponsor is Dr Peter Doran, Senior Lecturer in Medicine, University College Dublin, Dublin, Ireland.

## Discussion

Supplemental vitamin D appears to have potential as a therapeutic agent for MS; what is less clear is the optimal dose of vitamin D needed to effect change within the immune system. Our study is designed to address this question, and to add to the growing evidence of the safety and tolerability of high-dose vitamin D supplementation. As this is a phase II randomized, placebo-controlled trial with two distinct study groups and is adequately powered to meet the primary endpoint, it should be considered a high-quality trial aiming to provide class II evidence of the primary endpoint.

Existing guidelines on vitamin D supplementation are conservative, and refer mainly to bone health [33], with 4,000 IU considered the upper limit of tolerability, despite the fact that sun exposure can provide an adult with up to 20,000 IU per day [34]. Older adults are now advised to sustain 25(OH) D concentrations of greater than 75 nmol/l for optimal bone health and, in order to achieve that, they need to consume approximately 4,000 IU/day of vitamin D [18,35]. Levels greater than 100 nmol/l are needed to ensure that patients are receiving adequate immunomodulating doses of vitamin D. Based on previous dosing studies [18], the maximum serum 25(OH)D level expected with a daily dose of 10,000 IU is approximately 250 nmol/l, making both treatment doses safe and tolerable.

The availability of DMT and the earlier recognition and diagnosis of MS have a significant effect on trial design for the evaluation of new drugs, and this also raises ethical concerns. Most would feel that placebo-controlled studies of the RRMS population are unethical. This results in the need for large multicenter trials to achieve adequate numbers to power the study over longer time periods to assess for clinical endpoints. Thus, costs are generally prohibitive and really limits these studies to large pharmaceutical companies.

In Ireland, the UK, Australia, and New Zealand, patients with CIS are treated conservatively until there is evidence, on clinical or MRI grounds, of dissemination in time. Patients with CIS form a distinct population with a well-established natural history, who are not exposed to immunomodulatory therapy. Given the standard practice in Ireland, there is no ethical concern with regard to watchful withholding of treatment. Should patients convert to RRMS during the trial, they will be offered standard DMT. This study should add to the growing evidence of the immunomodulatory effects of high-dose supplemental vitamin D in demyelinating disease. We hope to show that vitamin D is a safe and effective therapy for patients with CIS.

## Trial status

This trial is active and has been recruiting from November 2012.

## Abbreviations

ARR: Annualized relapse rate; CIS: Clinically isolated syndrome; DMT: Disease-modifying therapy; MRI: Magnetic resonance imaging; MS: Multiple sclerosis; PBMC: Peripheral blood mononuclear cells; RCT: Randomized control trial; RRMS: Relapsing remitting multiple sclerosis; VDR: Vitamin D receptor.

## Competing interests

KO’C has received an educational grant from Biogen Idec and travel bursaries from Abbot, Teva, and Biogen Idec. SK has received educational grants from Abbot, Teva, and Biogen Idec. CMG has received honoraria and research funding from Biogen Idec, Bayer, Merck Serono, Novartis, Genzyme, and Teva. NT has received honoraria and research funding from Biogen Idec, Bayer, Merck Serono, Novartis, Genzyme, and Teva.MH served on a medical advisory board [BG00012] for Biogen Idec; serves on the editorial boards of the *Multiple Sclerosis Journal*, has received speaker’s honoraria from Biogen Idec, Bayer-Schering, and Novartis, and receives research support from Dystonia Ireland and the Health Research Board of Ireland. None of the other authors have any competing interests to declare.

## Authors' contributions

KO’C wrote the original and final draft of the manuscript, acts as study investigator, and is involved in patient recruitment, data collection, and analysis of all results. SK was involved in trial conception and design, and wrote the original protocol and ethics submission. KK is involved in patient recruitment, examination, and data collection. SJ is responsible for producing original source documents, maintaining site files, and dispensing of the study drug. OK is involved in patient recruitment and examination, and data collection. DM, EH, and RO’L are responsible for designing the imaging protocol and for acquisition and interpretation of all imaging carried out during the study. DO’S was involved in trial conception and design. CMK is the study pharmacist and is responsible for blinding, randomization, and dispensing of the study drug. LC is involved in recruitment and trial design. JF is responsible for all immunology outcome measures carried out as part of the trial, and for trial conception and design. JB is responsible for all endocrinology outcome measures carried out as part of the trial, and for trial conception and design. CW is responsible for adequately powering the study to meet the primary outcome, and for all statistical analysis as part of the trial. CMG is trial co-investigator, and is involved in trial conception and design, patient recruitment, and data collection and analysis. NT is trial co-investigator, and is involved in trial conception and design, patient recruitment, and data collection and analysis. MH is principal investigator of the study, and is responsible for trial conception and design, patient recruitment, data collection and analysis, and redrafting of this manuscript. All authors have reviewed and approved the above manuscript and suggested changes where appropriate.
